# Technologies evolution in robot-assisted fracture reduction systems: a comprehensive review

**DOI:** 10.3389/frobt.2023.1315250

**Published:** 2023-11-22

**Authors:** Wei Kou, Peiqing Zhou, Jihong Lin, Shaolong Kuang, Lining Sun

**Affiliations:** ^1^ Department of Mechanical and Electrical Engineering, Soochow University, Suzhou, China; ^2^ College of Health Science and Environment Engineering, Shenzhen Technology University, Shenzhen, China

**Keywords:** bone fracture reduction, virtual surgery, robot-assisted fracture reduction, preoperative planning, intraoperative registration, navigation, fracture reduction robot

## Abstract

**Background:** Robot-assisted fracture reduction systems can potentially reduce the risk of infection and improve outcomes, leading to significant health and economic benefits. However, these systems are still in the laboratory stage and not yet ready for commercialization due to unresolved difficulties. While previous reviews have focused on individual technologies, system composition, and surgical stages, a comprehensive review is necessary to assist future scholars in selecting appropriate research directions for clinical use.

**Methods:** A literature review using Google Scholar identified articles on robot-assisted fracture reduction systems. A comprehensive search yielded 17,800, 18,100, and 16,700 results for “fracture reduction,” “computer-assisted orthopedic surgery,” and “robot-assisted fracture reduction,” respectively. Approximately 340 articles were selected, and 90 highly relevant articles were chosen for further reading after reviewing the abstracts.

**Results and Conclusion:** Robot-assisted fracture reduction systems offer several benefits, including improved reduction accuracy, reduced physical work and radiation exposure, enhanced preoperative planning and intraoperative visualization, and shortened learning curve for skill acquisition. In the future, these systems will become integrated and practical, with automatic preoperative planning and high intraoperative safety.

## 1 Introduction

Bone fractures constitute the most common type of trauma and impose significant healthcare and economic burdens (Lee et al., 2014; Wu et al., 2021). Epidemiological studies have shown a global annual incidence of 9.0‰–22.8‰ for fractures (Tang et al., 2012). Surgical treatment of bone fractures is often necessary and considered a common and routine task (Kronman and Joskowicz, 2013). Traditionally, open surgery is performed to expose the fractured bones, followed by manual surgery. However, this method is often associated with a higher risk of infection and soft tissue failure, resulting in prolonged hospitalization, rehabilitation time, and substantial costs (Raabe et al., 2012; Dagnino et al., 2016a). Percutaneous techniques have been developed to alleviate these problems. These techniques use fluoroscopes to assist surgeons in manipulating fragments through small incisions, which reduces the risk of infection and allows for quicker recovery times. However, the outcomes of such methods rely heavily on surgeon experience, with the risk of increased radiation exposure and expensive revision operations to correct malposition (Du et al., 2015a; Zhao et al., 2020; Han et al., 2021).

**FIGURE 1:**
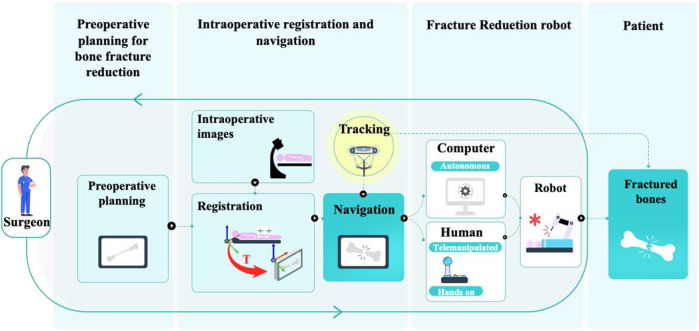
Typical RAFR surgery procedure.

The development of computer and robotic technologies has led to various robot-assisted fracture reduction (RAFR) systems to alleviate these problems. A typical surgical procedure using an RAFR system, illustrated in Figure 1, generally involves the following stages: preoperative planning for bone fracture reduction, intraoperative registration and navigation, and robotic fracture reduction (Dagnino et al., 2017a; Zhao et al., 2020). The earliest concept of an RAFR system dates back to 1995 (Bouazza-Marouf et al., 1995). Since then, organizations in Germany (Gosling et al., 2005; Westphal et al., 2006; Westphal et al., 2009a), New Zealand (Graham et al., 2006; Graham et al., 2008a), the United Kingdom (Raabe et al., 2012; Dagnino et al., 2017b; Dagnino et al., 2017c), China (Wang et al., 2014; Ge et al., 2022), and other countries (Joung et al., 2008; Maeda et al., 2008) have conducted relevant research. Compared with robot-assisted laparoscopic surgery (Gala et al., 2014; Longmore et al., 2020) and robot-assisted total hip or total knee arthroplasty (Panariello et al., 2019; Subramanian et al., 2019), which began development around the same time and have since matured to the point of commercialization, RAFR systems remain in the proof-of-principle stage. To date, no successful production has occurred in the capital market or clinical practice.

A comprehensive review of previous studies and clarification of the technological evolution process are necessary to identify technical solutions and future development trends to promote RAFR systems in the clinical and commercial fields. Several researchers have reviewed the techniques involved in RAFR systems. These studies provide valuable resources for researchers and practitioners in the field of RAFR systems. Zhao et al. (2020) provided a comprehensive overview of navigation and robotic systems, whereas Bai et al. (2019) focused on the structure of fracture reduction surgery robots and related assistive technologies. Moolenaar et al. (2022) and Jiménez-Delgado et al. (2016) described the main procedures and standard techniques used in computer-assisted fracture reduction preplanning, along with their advantages and disadvantages. However, the above reviews mainly focused on individual key technologies, system composition, and surgical stages. This review aims to assist future scholars in selecting appropriate research directions for clinical use. Specifically, it focuses on the technological evolution of surgical procedures that utilize RAFR systems.

To conduct a comprehensive literature survey, Google Scholar was searched for information on “fracture reduction,” “computer-assisted orthopedic surgery,” and “robot-assisted fracture reduction.” The search yielded 17,800, 18,100, and 16,700 results, respectively. Studies related to RAFR systems were selected, excluding those with the following characteristics:

(1) Robots were not used.
(2) The focus was not on reducing fractures (e.g., using robots to assist fixation, robot-assisted total hips, or total knee arthroplasty).

We selected approximately 340 articles with general relevance. After reading the abstracts, 90 highly relevant articles were selected for in-depth reading.

The rest of this paper is arranged as follows. Section 2 outlines the steps for implementing preoperative planning and the technologies involved in RAFR systems. Section 3 summarizes different registration methods and various types of navigation in RAFR systems. Section 4 outlines various types of robots and analyzes their strengths and weaknesses. Section 5 explores the challenges and development trends in RAFR systems. Finally, Section 6 summarizes the conclusions.

## 2 Technologies for preoperative planning of bone fracture reduction

**FIGURE 2:**
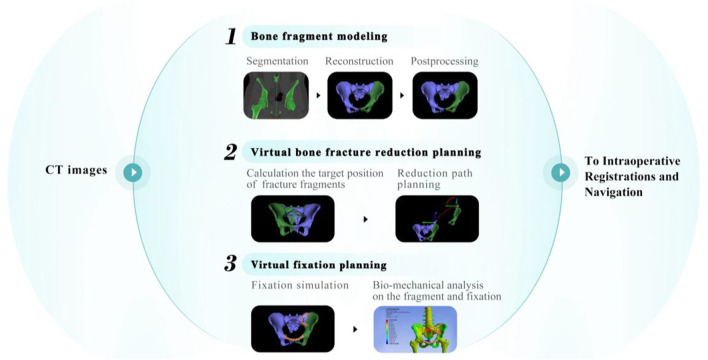
Overview of preoperative planning for RAFR.

Typical preoperative planning of bone fracture reduction involves the following steps: bone fragment modeling, virtual bone fracture reduction planning, and virtual fixation planning. An overview of these steps is shown in Figure 2.

### 2.1 Bone fragment modeling

The initial step in preoperative planning for bone fracture reduction is to create three-dimensional (3D) models of bone fragments that accurately represent the bones and fragments affected by injury. Computed tomography (CT) images preserve the actual anatomy, including depth information, and provide surgeons with detailed information about the fracture site. The modeling process consists of three stages: segmentation, reconstruction, and postprocessing. In the segmentation stage, CT images were used to distinguish bone tissue from other tissues. The four common segmentation methods are intensity-, edge-, region-, and registration-based approaches (Jiménez-Delgado et al., 2016). Various (semi-)automatic deep learning-based segmentation methods have recently been proposed (Wang et al., 2019; Liu et al., 2021). Depending on the intended use, bone fragment models can be represented as a volume, point cloud, or mesh during reconstruction. Finally, the models were subjected to postprocessing optimization for subsequent utilization. Jiménez-Delgado et al. (2016) extensively discussed these topics. The modeling process in RAFR is similar to that of other computer-assisted navigation systems in orthopedic surgery. However, this paper specifically focuses on techniques that are directly relevant to fracture reduction. Therefore, the details of the modeling process will not be extensively described here.

### 2.2 Virtual bone fracture reduction planning

The process of virtual bone fracture reduction typically involves two steps after bone fragment modeling: calculation of the target positions for fracture fragments and reduction path planning.

#### 2.2.1 Calculation of the target positions for fracture fragments

The goals of calculating the target positions of the fracture fragments are to determine their original positions and to calculate the rigid transformation between bone fragments to achieve accurate alignment in a virtual environment (Buschbaum et al., 2015; Suero et al., 2018). The common methods used in RAFR systems to accomplish this task include observation methods based on 3D computer-aided design (CAD) tools, fracture area matching, and registration based on templates. Table 1 summarizes all the studies that were reviewed regarding the calculation of target positions for fracture fragments.

**TABLE 1:** Types and characteristics of fracture fragment target position calculation methods in RAFR systems.

Types of methods	Typical features	Indications	References
Observation methods based on 3D CAD tools	Leap Motion and three-foot pedals; trial and error; reliance on the personal experience of surgeons	Joint fracture	Dagnino et al. (2015), Dagnino et al. (2016b)
Fracture area matching	Automatic calculation; cylinder fracture objects	Femur fracture	Winkelbach et al. (2003)
	Automatic calculation; broken arbitrary objects; time-consuming	Femur fracture	Winkelbach et al. (2004)
	Employment of lower matching quality	Femur fracture	Winkelbach et al. (2006), Westphal et al. (2009a)
Registration based on templates	Matching the 3D image of the broken side with the mirrored unfractured side; reliance on mirror symmetrical relations	Femur fracture/diaphyseal fracture/pelvic fracture	Tang et al. (2012); Hu et al. (2013); Wang et al. (2014), Du et al. (2015b); Kim and Ko, (2019), Wang et al. (2016), Zhao et al. (2022a); Zhao et al. (2022b); Ge et al. (2022)

**FIGURE 3:**
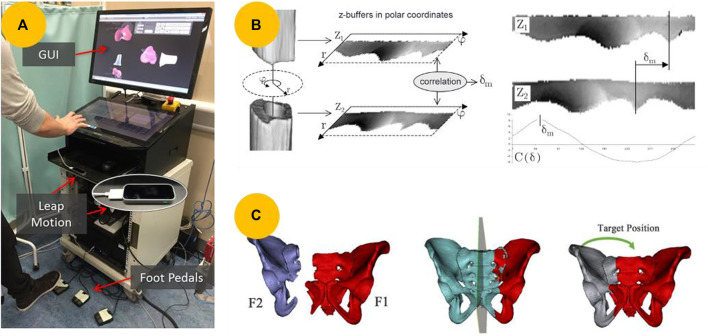
Methods of calculating the target positions of fracture fragments. **(A)** Interacting with 3D models using a Leap Motion and three-foot pedals to manually reduce fractures (Dagnino et al., 2016b). **(B)** Maximizing the matching of fracture surfaces between two cylindrical fracture segments using a z-buffer algorithm (Winkelbach et al., 2003). **(C)** Virtual reduction of pelvic fracture using the contralateral mirror template (Zhao et al., 2022b).

The earliest and most common method of calculating the target positions of fracture fragments is observation methods based on 3D CAD tools. Surgeons manually manipulate fracture fragments using interfaces such as mice, haptic systems, and virtual reality (VR) environments while observing screens to judge whether the target positions have been achieved based on their experience (Fornaro et al., 2008; Fornaro et al., 2010). Dagnino et al. (Dagnino et al., 2015; Dagnino et al., 2016b) utilized a dedicated graphical user interface that allows preoperative visualization and manipulation of fragments using Leap Motions and three-foot pedals, as shown in Figure 3A. However, observation using 3D CAD tools is only suitable for simple fractures, requires trial and error, and relies on surgeon experience. Further, aligning bones using this approach remains challenging.

Reducing comminuted fractures using observation methods is nearly impossible for the above reasons. Instead, fracture reduction can be considered a solution to the 3D puzzle problem (Winkelbach et al., 2003). Scholars have proposed an automatic virtual fracture reduction method that aims to match fracture areas and maximize the matching fracture surfaces of two fracture segments in contact. In 2003, Winkelbach et al. (2003) proposed an approach to detect cylinder axes and compute relative transformations between fragments, as illustrated in Figure 3B. However, this method is limited to reducing cylindrical fracture objects and is suitable only for long bone fracture reduction. To address this issue, Winkelbach et al. (2004) proposed an efficient pairwise surface-matching approach for reconstructing broken objects. In a subsequent study, they improved this method and introduced a random sample matching algorithm (Winkelbach et al., 2006). In 2009, Westphal et al. (2009a) developed a telemanipulator RAFR system that utilizes this algorithm, achieving high reduction accuracies for complex fractures. Some studies have also matched the fracture lines extracted from the affected areas to obtain the target poses of the fracture fragments (Buschbaum et al., 2015; Buschbaum et al., 2017). However, defining the fracture lines is challenging. Additionally, the methods described above rely on the unique features of fractured bones and thus can only be applied to certain types of fractures.

Numerous automated registration methods based on contralateral mirroring templates have been suggested to determine the target positions of the fracture fragments for various types of fractures. Tang et al. (2012) developed a hexapod reduction system that automatically reduces a broken leg by matching a 3D image of the fracture with a mirrored 3D image of the unfractured leg. Wang et al. (2014), Du et al. (2015b), and Kim and Ko (2019) applied this method to RAFR systems to reduce diaphyseal fractures. Compared with diaphyseal fractures, pelvic fractures have complex anatomical features, and their geometric parameters can vary among individuals. Wang et al. (2016) proposed a reduction reference based on physical symmetry and a virtual plane. However, this method is impractical, as it requires multiple manual steps. To solve this problem, Zhao et al., 2022a) proposed a method called pelvic symmetry reduction for automatic pelvic fracture reduction planning, which replaces multiple manual operations, as shown in Figure 3C. In a subsequent study, Zhao et al. (Zhao et al., 2022b; Ge et al., 2022) integrated this method into an automatic reduction algorithm for robotic pelvic fracture reduction systems. However, these methods, which rely on mirror-symmetry relations, are not applicable in cases where both sides are broken, or there are natural shape differences between them (Han et al., 2021). Therefore, some researchers have used statistical shape models (SSMs) as templates to reduce fractures virtually in computer-assisted orthopedic surgery (Albrecht and Vetter, 2012; Ead et al., 2021; Han et al., 2021; Lu et al., 2023).

Currently, no unified method exists for calculating the target position of fracture fragments. Observation methods based on 3D CAD tools and fracture area matching are suitable only for certain fracture types. The mainstream method adopted was registration based on contralateral mirroring templates. However, this method is not applicable in all situations. As the number of fracture types and training model samples increases, registration based on SSM templates may be a solution.

#### 2.2.2 Reduction path planning

After the surgeon has calculated the initial position of the fracture fragments, an effective reduction path is planned to move them to the target position. To plan an optimal reduction path, several factors must be considered, including minimizing the force of the muscle resistance, keeping the path as short as possible, and avoiding potential bone collisions. These considerations ensure a gentle and safe reduction path while minimizing soft tissue damage. Table 2 summarizes all the reviewed studies regarding path planning for fracture reduction.

**TABLE 2:** Types and characteristics of path planning methods in RAFR systems.

Types of reduction path planning	Path search methods	Collision avoidance methods	Reduction force estimation methods	Indications	References
Manual/Semi-automatic	Manual	Observation	Personal experience	Joint fracture	Dagnino et al. (2016b), Dagnino et al. (2017b), Dagnino et al. (2017c)
	Shortest linear path	Reduce to the location offset along the proximal fragment shaft		Femoral fracture	Ye and Chen (2009a)
	Shortest linear path	Observation		Pelvic fracture	Zhao et al. (2022b)
	Cost function	Observation	Minimize the amount of distraction	Femoral fracture	Westphal et al. (2009a), Westphal et al. (2009b)
	Fracture classification	Observation	Minimize the amount of distraction	Femoral fracture	Du et al. (2015a), Du et al. (2015b)
	Varying the sequences of movements or movement patterns	Observation	Personal experience	Femoral fracture	Buschbaum et al. (2015)
Automatic	Modified A* algorithm	Point-in-polyhedron and ray-cast method	OpenSim model	Femoral fracture	Buschbaum et al. (2017)
	Improved 3D A* algorithm	Intersection test	Length of the main muscle	Femoral fracture	Xu et al. (2022)
	Improved RRT* algorithm	Cylindrical and spherical bounding boxes technique	—	Tibial fracture	Li et al. (2022)
	Augmented A* algorithm	Combination of the intersection test with the octree spatial division algorithm and bounding box technique	—	Pelvic fracture	Pan et al. (2023)
	3D-OPSF A* algorithm	Combination of spatial segmentation, spatial overlap testing, and bounding box techniques	OpenSim model	Pelvic fracture	Chen et al. (2023)

Note: “/” indicates not applicable.

Manual planning based on anatomical knowledge is the simplest and most common method of planning a reduction path. Dagnino et al. (Dagnino et al., 2016b; Dagnino et al., 2017b; Dagnino et al., 2017c) manually planned desired paths for robot-assisted joint fracture reduction. However, this approach is time-consuming and heavily reliant on the personal skills and experience of the surgeon. To simplify and expedite the planning process, some scholars have planned reduction paths based on the shortest possible route, prioritizing keeping the path as short as possible. Ye and Chen (2009a) developed a path planning algorithm for femoral shaft fractures based on finding the shortest linear path, while Zhao et al. (2022b) developed a similar algorithm for pelvic fractures. However, to ensure gentle and safe reduction, the path must be kept as short as possible, and the reduction forces must be minimized.

**FIGURE 4:**
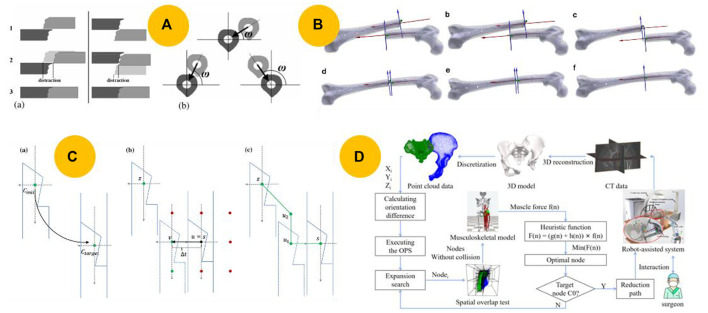
Various reduction path planning methods. **(A)** Minimizing distractions by selecting a favorable approach direction based on a cost function (Westphal et al., 2009b). **(B)** Reduction path consisting of manually controlled individual reduction movements (Buschbaum et al., 2015). **(C)** Automatic reduction path planning in the frontal plane based on the modified A* algorithm (Buschbaum et al., 2017). **(D)** Automatic planning of the reduction path with the least muscle resistance using 3D-OPSF A* (Chen et al., 2023).

Generally, the reduction procedure for femoral fractures involves distraction, rotation, and translation. Westphal et al. (Westphal et al., 2009a; Westphal et al., 2009b) developed a cost function that considered both the amount of distraction and rotation to determine the optimal approach direction for the fracture region, as illustrated in Figure 4A. Similarly, Du et al. (2015a) developed a new fracture classification system that uses the overlap length after a fracture. In a subsequent study, Du et al. (2015b) used this classification system and a modified hexapod device to reduce closed diaphyseal fractures. Buschbaum et al. (2015) further divided the femoral fracture reduction procedure into six movement patterns, as shown in Figure 4B.

The manual and semi-automatic methods described above can help surgeons plan reduction paths as short as possible to avoid collisions. However, because of the subjective nature of surgeons, guaranteeing that the planned path is the optimal reduction path with the shortest reduction path and minimal reduction force is difficult. In addition, these methods are time-consuming.

Several studies have focused on automatic reduction path planning to address these issues. The A* algorithm is typically used for path searching in fracture reduction owing to its high efficiency. Musculoskeletal models have been developed to predict or monitor the forces produced during reduction procedures, including hip fractures (Joung et al., 2011), pelvic fractures (Elabjer et al., 2009; Liu et al., 2022; Kou et al., 2023), and femur fractures (Graham et al., 2008a; Li et al., 2015a). These models serve as the basis for the quantitative reduction force analysis of various reduction paths. Building on their earlier study on reduction path planning, Buschbaum et al. (2017) proposed an automatic path planning method based on a modified A* algorithm, as shown in Figure 4C. However, this method searches for a collision-free path with minimal force while only requiring translational movement. Inspired by this research, Xu et al. (2022) proposed an improved 3D A* algorithm to determine the femoral fracture reduction path in space. Their algorithm utilized real-time updates of the point cloud to detect collisions and calculated the lengths of major muscles to prevent soft tissue injury.

The methods described above apply only to lower-extremity fractures. Pan et al. (2023) proposed an augmented A* algorithm, 3D-OPS A*, to automatically plan reduction paths for more complex pelvic fractures. However, this approach does not guarantee a minimal resistance force during the reduction. In a further study, Chen et al. (2023) introduced a method called 3D-OPSF A* that searches for the path of least muscle resistance, as shown in Figure 4D.

During physical reduction procedures, a reasonable reduction path can help avoid collisions and prevent soft-tissue injuries. Researchers have increasingly focused on automatically reducing path planning in recent years, with most studies concentrating on path search methods. Whether manual, semiautomatic, or automatic methods, such as the A* or RRT* algorithm (Li et al., 2022), are used, the primary goal is to find the shortest path while avoiding collisions. However, fracture reduction is a complex problem involving the surrounding soft tissues, which induces resistance during the reduction procedure. This makes the situation more challenging, and the shortest path may not necessarily be optimal for reducing the force. Some musculoskeletal models can predict and manage the resistance force; however, further research will require a comprehensive model that fits the RAFR system.

### 2.3 Virtual fixation planning

After virtually reducing a fracture, the next steps involve placing virtual fixation devices for stabilizing the fracture and conducting a biomechanical analysis of the fragment and fixation. Moolenaar et al. (2022) discussed this topic in detail and provided insights into various types of fixation devices and their effectiveness. Therefore, the details of virtual fixation planning will not be extensively described here.

## 3 Technologies for intraoperative registration and navigation

An intraoperative registration process should be conducted to ensure optimal implementation of preoperative planning by navigation, providing the geometrical relationship between the surgical object (SO) and virtual object (VO) (Nolte and Beutler, 2004; Zheng et al., 2007). According to the classification of Nolte and Beutler (2004), the navigation used in RAFR systems can be categorized into three types based on the virtual representation of SO: 2D fluoroscopy-based navigation, 3D fluoroscopy-based navigation, and CT-based navigation. In essence, navigation relies on advancements in registration technology. The aforementioned navigation can be summarized as follows in terms of registration technologies. Table 3 lists all reviewed studies on intraoperative registration and navigation in RAFR systems.

**TABLE 3:** Types and characteristics of intraoperative navigation and registration technologies in RAFR systems.

Types of navigation	Using preoperative CT	Intraoperative data	Registration techniques	Typical features	Indications	References
2D fluoroscopy-based navigation	NO	2D fluoroscopic images	No preoperative images registration	Last X-ray image is overlaid with the moving bone axis	Femur shaft fracture	Westphal et al. (2006)
3D fluoroscopy-based navigation	NO	Iso C 3D		Surgical planning is conducted and implemented in the operation room	Femur fracture/femoral head fracture/femoral shaft fractures/intra-articular joint fractures	Warisawa et al. (2004), Mitsuishi et al. (2005), Westphal et al. (2008); Westphal et al. (2009a); Westphal et al. (2009b); Westphal et al. (2009b); Oszwald et al. (2009), Raabe et al. (2012)
CT-based navigation	NO	CT images		Special design of orthopedic pins	Intra-articular lower-limb fracture	Dagnino et al. (2017b), Dagnino et al. (2017c)
	YES	2D fluoroscopic images	2D/3D image-based registration	Conventional	Long bone fracture	Kim et al. (2016)
	YES	2D fluoroscopic images		CT-scan is conducted before inserting the orthopedic pins; a custom-made fiducial marker	Joint fracture	Dagnino et al. (2017b), Dagnino et al. (2017c)
	YES	Locations of fiducial markers	3D/3D feature-based registration	Paired–point matching; fiducial markers	Femur fracture	Lee et al. (2013), Kim and Ko, (2019)
	YES	Locations of fiducial markers		Micron tracker	Diaphyseal fracture	Li et al. (2015b), Li et al. (2016)
	YES	Digitized anatomical points		Match landmarks on the preoperative CT and the spatial coordinates	Pelvic fracture	Wu et al. (2020)
	YES	Iso C 3D	Image fusion-based registration	Non-rigid ICP algorithm; high-precision registration	Pelvic fracture	Zhao et al. (2022b), Ge et al. (2022), Shi et al. (2021)

### 3.1 No preoperative images registration techniques

**FIGURE 5:**
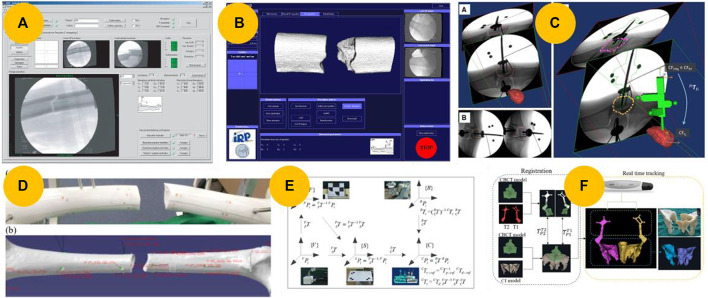
Intraoperative registration and navigation technologies. **(A)** User interface for a navigation system based on 2D fluoroscopy (Westphal et al., 2006). **(B)** User interface for navigations based on 3D fluoroscopy (Westphal et al., 2008). **(C)** An image registration framework enables the registration of pre-operative CT images and intra-operative fluoroscopic images of a fractured bone using a custom-made fiducial marker (Dagnino et al., 2017b; Dagnino et al., 2017c). **(D)** Twenty-four fiducial markers attached to a femur mockup for paired-point matching (Lee et al., 2013). **(E)** Transformations of coordinate systems among the model, robot, surgical operation, and visual coordinate systems (Li et al., 2015b). **(F)** Real-time 3D navigation enabled by registering the cone beam computed tomography (CBCT) and CT models (Shi et al., 2021).

Intraoperative fluoroscopy is a conventional and useful tool for observing closed reductions. The transformation from a C-arm fluoroscope into VO can be determined by modeling the cone-beam X-ray projection of the C-arm and compensating for image distortions and C-arm deformations (Hofstetter et al., 1999; Nolte and Beutler, 2004). Based on this principle, Westphal et al. (2006) developed a surgical telemanipulator with a navigation system. In their system, only the defined bone axis moves in the image when a surgeon moves the robot, as shown in Figure 5A. However, this can be improved by enabling more intuitive interaction with 3D imaging.

Siemens Medical Solutions introduced SIREMOBIL Iso-C 3D in 1999, the first mobile C-arm-type intraoperative 3D imaging device to capture CT-like volume data (Nolte and Beutler, 2004; Zheng et al., 2007). The registration principle aligns with using intraoperative fluoroscopy, but Iso-C 3D simplifies the process of intraoperative registration. Based on their experience with telemanipulated fracture reductions using 2D X-ray imaging, Westphal et al. (Westphal et al., 2008; Westphal et al., 2009a; Westphal et al., 2009b; Oszwald et al., 2009) further improved and developed a RAFR system. The system, shown in Figure 5B, utilizes intraoperative 3D imaging. This advancement has resulted in excellent accuracy, intuitive operation, and improved efficiency. Utilizing this registration method, scholars also developed RAFR systems for femur fractures (Warisawa et al., 2004), femoral head fractures (Mitsuishi et al., 2005), and intra-articular joint fractures (Raabe et al., 2012). Normally, the image quality of Iso 3D was not as good as that of CT. Dagnino et al. (Dagnino et al., 2015; Dagnino et al., 2016b) used CT images and specially designed orthopedic pins to perform intraoperative registration.

However, the methods mentioned above obtain images intraoperatively, which leads to time-consuming surgical planning that must be done during the surgery. In fact, preoperative CT scans are routinely performed, enabling surgical planning can be conducted in advance. In most cases, intraoperative data typically need to be acquired from the fracture region using either a fluoroscope or dynamic referencing bases (Zheng et al., 2007; Dagnino et al., 2017c). Subsequently, a 2D/3D or 3D/3D image-based registration method will be employed for intraoperative registration.

### 3.2 2D/3D image-based registration techniques

The registration techniques for 2D/3D registration can be classified into two main types: feature- and intensity-based techniques (Zheng et al., 2007). Feature-based methods require accurate extraction of bone contours from fluoroscopic images, whereas intensity-based methods rely on selecting an appropriate similarity measure. Due to their low computation cost, feature-based methods are commonly used in most RAFR systems for 2D/3D feature-based registration. Kim et al. (2016) proposed a robot-assisted long bone fracture reduction system that performed two 2D/3D feature-based registrations. Maintaining a sterile environment is crucial for performing a preoperative CT scan after orthopedic pin insertion, but it can be challenging in clinical practice. To resolve this problem, Dagnino et al. (Dagnino et al., 2017b; Dagnino et al., 2017c) proposed a redesigned navigation system that was improved using an image registration framework to implement the RAFR system in an actual surgical environment, as shown in Figure 5C. 2D/3D feature-based registration techniques are error-prone and inaccurate. Obtaining accurate contours of fragments from fluoroscopic images is difficult in clinical practice because of fragment overlap and the influence of other tissues.

### 3.3 3D/3D image-based registration techniques

A more effective method to improve the registration accuracy further is to use artificial objects such as pins or percutaneous marks for paired-point matching (Zheng et al., 2007). Lee et al. (2013) and Kim and Ko (2019) proposed a navigation system for robot-assisted femoral fracture reductions, utilizing 24 fiducial markers attached to the femur mockup before CT scanning, as shown in Figure 5D. However, the requirement for fiducial markers to be implanted on a broken femur goes against the goal of minimizing invasiveness.

Li et al. (Li et al., 2015b; Li et al., 2016) proposed a visual servo-based teleoperation robot system that does not require implanted artificial markers, as shown in Figure 5E. Wu et al. (2020) established the positional correspondence between 3D models and the intraoperative pelvis through registration, matching spatial coordinates of manually extracted feature points on the pelvic bone using intraoperative fluoroscopy images and corresponding feature points on preoperative CT. Some scholars also used intelligence algorithms to automatically extract feature points, aiming to enhance registration accuracy and reduce registration time (Seim et al., 2009; Wang et al., 2022). The aforementioned method does not require additional markers implantation and meets the minimally invasive surgery requirements. However, the registration process is still complex and time-consuming.

### 3.4 Image fusion-based registration techniques

To combine the advantages of high image quality provided by a standard CT scan with the simplicity of registration using Iso-3D, some scholars have utilized image fusion-based registration techniques for navigation. Shi et al. (2021), Zhao et al. (2022b), and Ge et al. (2022) proposed a 3D navigation system to assist with pelvic fracture reduction using robots. The system uses preoperative CT, intraoperative CBCT, and the nonrigid ICP algorithm for registration, as shown in Figure 5F.

3D navigation has become mainstream in RAFR systems, with the key problem being registration. Several approaches have been suggested to address this problem. However, Iso-3D imaging is rarely used in clinical practice because of constraints in the operating room, such as cost and space limitations. Paired-point matching often involves additional trauma and infection risks, whereas 2D/3D feature-based registration, which projects 3D geometry onto a 2D X-ray, is difficult and prone to errors. In the future, the registration process should aim to be minimally invasive, using less radiation and simpler processes while achieving higher precision.

## 4 Technologies for fracture reduction robot

Finally, the robot performs the physical reduction either through telemanipulation/hands-on by the surgeons or through automatic control by navigation. The structure and installation form determine the complexity of the surgical procedure. Various robotic systems have been developed to satisfy fracture reduction requirements, including workspace, output force, safety issues, and accuracy. These robots can be divided into two types according to their structure: robots based on external fixators, robots for distraction and reduction. Table 4 summarizes all the reviewed studies regarding robots for fracture reduction.

**TABLE 4:** Types and characteristics of fracture reduction robots in RAFR systems.

Types of reduction robot	Human–robot interaction modes	Typical features	Indications	References
Robots based on external fixators	Telemanipulated	RX 130; a force/torque sensor	Femur fracture	Füchtmeier et al. (2004)
	Telemanipulated	RX90; a joystick with force feedback; a force/torque sensor	Femur shaft fracture	Westphal et al. (2006); Westphal et al. (2008); Westphal et al. (2009a); Westphal et al. (2009b)
	Hands-on	HA006; two force/torque sensors	Long bone fracture and femur fracture	Kim et al. (2016); Kim and Ko, (2019)
	Telemanipulated	A force/torque sensor; two master–slave modes	Pelvic fracture	Wu et al. (2020)
	Autonomous	UR16e; traction device	Pelvic fracture	Shi et al. (2021), Zhao et al. (2022a), Ge et al. (2022)
Robots for distraction and reduction	Autonomous	Unilateral external fixator; accurate execution	Femur fracture	Kim and Lee, (2004); Kim and Lee, (2006)
	Autonomous	Hexapod robot external fixator; load measurement capabilities	Femur fracture	Seide et al. (2004a)
	Autonomous	Stewart platform; acts as an external fixator after the reduction	Long-bone fracture	Majidifakhr et al. (2009), Tang et al. (2012)
	Autonomous/hands-on	Traction boot; a force/torque sensor	Femur fracture/femoral head fracture	Warisawa et al. (2004), Mitsuishi et al. (2005), Maeda et al. (2005); Maeda et al. (2008)
	Autonomous	Two mechanical failsafe units; a force/torque sensor	Hip fracture	Joung et al. (2008); Joung et al. (2010)
	Telemanipulated	Cuff-type reduction unit	Femur shaft fracture	Sun et al. (2015), Zhu et al. (2017)
	Telemanipulated/semi-autonomous/autonomous	Emulate the approach of traditional clinical treatment; a force/torque sensor	Femur fracture/tibia fracture	Fu et al. (2004), Kong et al. (2006); Wang et al. (2006); Zhi-jiang et al. (2006)
	Telemanipulated/autonomous	An active force/position controller	Long bone fracture	Mukherjee et al. (2005), Graham et al. (2006); Graham et al. (2008b)
	Telemanipulated	Disk platform and a two-thirds circular ring	Femoral shaft fracture	Wang et al. (2013)
	Telemanipulated/autonomous	Linear movements; A force/torque sensor	Femur fracture	Ye and Chen, (2009b); Ye et al. (2012), Wang et al. (2009); Song et al. (2010)
	Autonomous	Removable series-parallel mechanism	Long bone fracture	Wang et al. (2014)
	Autonomous	Reduction and positioning units;hydraulic drive; a force/torque sensor	Diaphyseal fracture	Li et al. (2014), Du et al. (2015b), Li et al. (2015b)
	Telemanipulated	Master–slave	Diaphyseal fracture	Li et al. (2016)
	Autonomous	Three automated spatial parallel manipulators	Intra-articular joint fracture	Raabe et al. (2012)
	Autonomous	Open-loop and closed-loop position controllers; a force/torque sensor	Joint fracture	Dagnino et al. (2016b); Dagnino et al. (2016c)
	Autonomous	Four motorized actuators	Intra-articular fracture	Dagnino et al. (2016a)
	Autonomous	Two robotic fracture manipulators and two carrier platforms	Joint fracture	Dagnino et al. (2017b); Dagnino et al. (2017c)
	Telemanipulated	Hexpod robot and two series manipulators	Pelvic fracture	Bignardi et al. (2018)

### 4.1 Robots based on external fixators

#### 4.1.1 Unilateral external fixators

**FIGURE 6:**
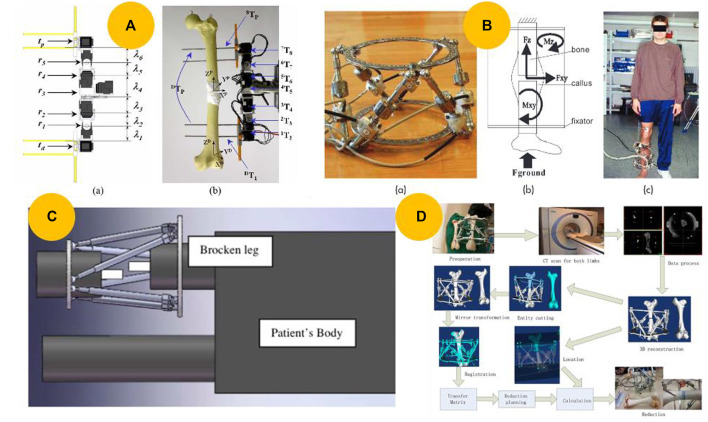
Robots based on external fixators. **(A)** Prototype of a motorized external fixation robot for fracture surgery (Kim and Lee, 2006). **(B)** Hexapod robot external fixator (Seide et al., 2004a). **(C)** Schematic for implementing an external fixator at the fracture site in the operation room (Majidifakhr et al., 2009). **(D)** Computer-assisted, motorized hexapod-based fracture reduction system (Tang et al., 2012).

Unilateral external fixators are commonly used to stabilize long bone segments after a fracture or to correct deformities resulting from fractures (Chao and Hein, 1988). These methods were employed even before other methods of fracture reduction became available. However, inaccurate surgical planning can result in poor clinical outcomes. To address this issue, Kim et al. (Kim and Lee, 2004; Kim and Lee, 2006) developed a prototype robotic unilateral external fixator using the Dynafix^®^ (EBI Medical, United States) external fixation system to correct bone deformities, as shown in Figure 6A. Although robots based on unilateral external fixators are low-cost and have simple structures, the serial structure often leads to low accuracy.

#### 4.1.2 External ring-type fixators

In 1965, Stewart introduced the hexapod, which was later modified by Taylor in the United States to develop the Taylor spatial frame (Stewart, 1965; Taylor, 2008), and by Seide in Germany to develop a precision hexapod external fixator (Seide et al., 2004b). In fracture treatment, the fixator is applied quickly and then slowly adjusted over the next few days while the patient remains in bed to avoid further soft tissue damage. In their subsequent study, Seide et al. (Seide et al., 2004a; Seide et al., 2004b) developed a motorized fracture robot and a 6-DOF measuring fixator by adding electromotor elements and six uniaxial force-measuring elements to a manually controlled fixator, as shown in Figure 6B.

In contrast to the gradual reduction method mentioned above, which requires a few days, scholars have presented ring-type external devices for operating room procedures. Majidifakhr et al. (2009) designed an external fixator to reduce fractures in the operating room, as illustrated in Figure 6C. Based on this concept and navigation technology, Tang et al. (2012) developed a computer-assisted motorized hexapod-based fracture reduction system, as illustrated in Figure 6D.

One key benefit of this type of robot is its ability to achieve highly precise fracture reduction automatically. In addition, it is small, lightweight, and easy to use in an operating room. However, it is only suitable for long bone fractures, and its load capability is insufficient owing to the power limitations of the motor. Furthermore, DC motors occupy excessive space, risking jamming and limiting the operation by the surgeon (Du et al., 2015b). Therefore, more acceptable robots must be developed.

### 4.2 Robots for distraction and reduction

#### 4.2.1 Serial kinematic distraction and reduction robot

**FIGURE 7:**
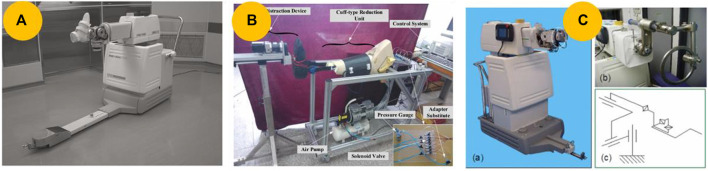
Serial kinematic distraction and reduction robot. **(A)** FRAC-Robo, which is based on a serial kinematic distraction device and performs reduction by using a traction boot (Maeda et al., 2008). **(B)** Cuff-type robot for long bone fracture reduction (Sun et al., 2015). **(C)** Robot modified with a customized jig connected to the fracture fragments (Joung et al., 2008).

An orthopedic traction table is commonly used conventional manual reduction equipment. Based on the working principle, some researchers have proposed robots that utilize parallel or serial kinematic distraction devices to assist with fracture reduction. In 2004, Warisawa et al. (2004) developed a robot with a motorized traction device using parallel kinematics to assist in reducing femoral fractures. Mitsuishi et al. (2005) expanded its applications to femoral head fracture reduction, and Maeda et al. (Maeda et al., 2005; Maeda et al., 2008) further improved and developed a robot called FRAC-Robo, as shown in Figure 7A. In contrast to the aforementioned motorized robots, Sun et al. (2015) proposed a noninvasive robotic system that utilizes pneumatic technology to reduce femur shaft fractures, as shown in Figure 7B. The robots mentioned above indirectly contact the femur for noninvasive fracture reduction. However, this approach makes it difficult to align fractures precisely. To address this issue, Joung et al. (2008) inserted two orthopedic pins into a bone fragment and connected the fragment to a customized jig, as shown in Figure 7C. However, because of the characteristics of serial kinematics, achieving a large reduction in force often results in bulky and heavy devices, making movements difficult and adding significant pressure to an already constricted operating room space.

**FIGURE 8:**
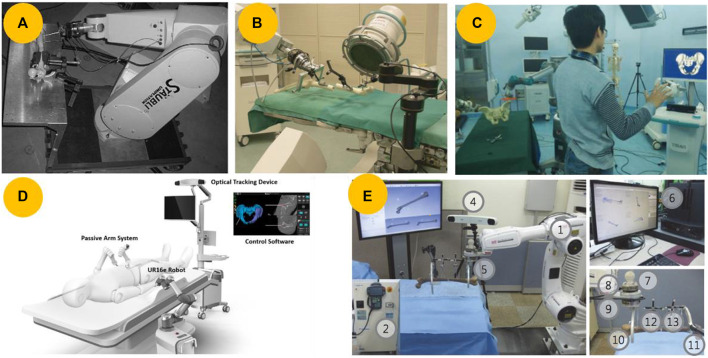
Robots based on serial manipulators. **(A)** Stäubli robot (Model RX130) modified as a reduction robot, named RepoRpbo (Füchtmeier et al., 2004). **(B)** Surgical tele-manipulator for femur shaft fracture reduction utilizing the clean-room version of an industrial robot (RX 90, Stäubli Tec-Systems, Faverges, France) (Westphal et al., 2006). **(C)** Robot-assisted pelvic fracture reduction robot utilizing a TiRobot® orthopedic robot system (Wu et al., 2020). **(D)** Robot-assisted pelvic fracture reduction system utilizes a 6-DOF robotic manipulator (UR16e) built on a mobile platform (Ge et al., 2022). **(E)** Hands-on robotic system utilizing a robot manipulator (HA006, Hyundai Heavy Industries Co., Ulsan, Korea) (Kim et al., 2016).

A few robots use robotic arms adapted from industrial robots to address the requirements of various types of fractures. This type of serial arm can be used to manipulate medical devices directly and provide large working spaces. In 2004, Füchtmeier et al. (2004) modified a Stäubli RX130 robot called RepoRobo, as shown in Figure 8A. To investigate the potential benefits of robot assistance in fracture reduction further and gather evidence for future research, Westphal et al. (2006) introduced a surgical tele-manipulator system that uses a Stäubli RX90 cleanroom robot for long bone fracture reduction, as shown in Figure 8B. While this system may be accurate for simple fracture types, it may not provide accurate reduction for complex fractures that do not have a direct connection between fragments. To address this issue, Westphal et al. (Westphal et al., 2008; Westphal et al., 2009a; Westphal et al., 2009b) added 3D navigation and automated preoperative planning to their system.

The robots mentioned above can assist with long bone or femur fractures. For pelvic fractures, Wu et al. (2020) utilized a 6-DOF serial manipulator and a robot-assisted traction device to provide flexible operation, as depicted in Figure 8C. Shi et al. (2021), Zhao et al. (2022b), and Ge et al. (2022) used a commercially available 6-DOF robotic manipulator, which was mounted on a mobile platform as depicted in Figure 8D.

Surgeons may want to intervene during robotic surgery to guide robots safely and accurately. To facilitate this intervention, some surgical robotic systems incorporate hands-on robotics in addition to telemanipulation or autonomous operations. Kim et al. (Kim et al., 2016; Kim and Ko, 2019) proposed a hands-on robotic system that utilized a bespoke robot manipulator with 6-DOF force feedback to reduce long-bone fractures, as seen in Figure 8E. In contrast to common robots that use only one force/torque sensor, this particular robot has two sensors attached to the end of its robotic arm.

This type of robot is based on a serial manipulator and offers several advantages, including a large workspace, high maneuverability, and ease of obtainment (Ye et al., 2012). However, it was originally designed for industrial use, and a large workspace can result in collisions in the operating room, leading to safety issues. Furthermore, owing to the mass of the following links, each link becomes burdened, resulting in low position accuracy and payload-to-weight ratio. This low ratio leads to heavy weight and large volume, resulting in high costs, crowded operating rooms, and poor portability.

#### 4.2.2 Parallel kinematic distraction and reduction robot

**FIGURE 9:**
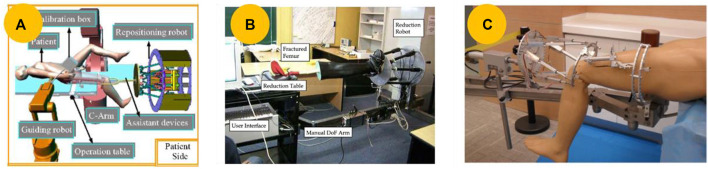
Parallel kinematic distraction and reduction robot. **(A)** Repositioning robot for the robot-assisted bone-setting system (Zhi-jiang et al., 2006). **(B)** Prototype for reducing long bone fractures, which consists of a 6-DOF parallel platform mechanism and a reduction table (Graham et al., 2008b). **(C)** Parallel manipulator robot on a traction table (Wang et al., 2013).

To maximize the utilization of valuable operating room space and facilitate transport, some researchers have attempted to use parallel kinematic distraction devices to reduce fracture. Fu et al. (2004) and Du et al. (Kong et al., 2006; Wang et al., 2006; Zhi-jiang et al., 2006) presented a flexible parallel robotic system that assisted surgeons in performing bone-setting operations, as illustrated in Figure 9A. Mukherjee et al. (2005) and Graham et al. (Graham et al., 2006; Graham et al., 2008b) introduced a RAFR system that employed a flexible parallel robot with an active force/position control algorithm to assist in the reduction of long bone fractures, as shown in Figure 9B. To apply the RAFR system to operating scenarios, Wang et al. (2013) developed a robot with a parallel manipulator robot mounted on a traction table, as shown in Figure 9C. Traditional traction devices typically have lower accuracy and are limited to providing force along a single axis, making realignment challenging. In contrast, parallel kinematic distraction devices can provide sufficient DOFs, reduction forces, and compact volumes, making it possible to correct alignment and malrotation adequately (Graham et al., 2006). However, owing to the limited workspace of the parallel mechanism, this type of robot is only suitable for lower extremity fractures, thereby limiting its clinical applicability.

#### 4.2.3 Serial-parallel hybrid kinematic distraction and reduction robot

Robots that use serial mechanisms often offer significant benefits, such as a wide range of motion, flexibility, and the ability to move around in large spaces. Robots that utilize parallel mechanisms, on the other hand, tend to have strong lifting capabilities relative to their weight, high rigidity, and precise positioning (Sun et al., 2015; Zhao et al., 2020). Scholars have proposed robots based on serial-parallel hybrid mechanisms for various fracture reductions to balance accuracy and workspace.

**FIGURE 10:**
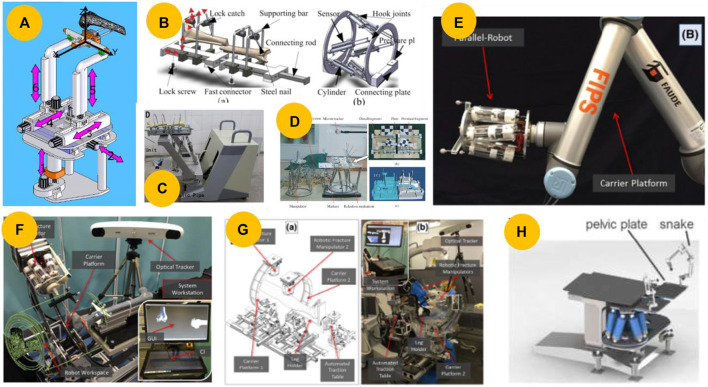
Robots based on serial–parallel hybrid mechanism. **(A)** Serial–parallel hybrid reduction robot with six independent linear actuators and a stiff end-effector (Ye et al., 2012). **(B)** Removable serial–parallel hybrid reduction robot (Wang et al., 2014). **(C)** Closed diaphyseal fracture reduction robot consisting of a reduction unit and a position unit (Du et al., 2015b). **(D)** Prototype of a master–slave teleoperation robot (Li et al., 2016). **(E)** Robot comprises a carrier platform (UR10) and a 6-DOF parallel robot for joint fracture surgery (Dagnino et al., 2016c). **(F)** Improved robot in which the UR10 is replaced with four motorized actuators (Dagnino et al., 2016a). **(G)** Joint fracture reduction robot based on two robotic fracture manipulators attached to two carrier platforms (Dagnino et al., 2017c). **(H)** Prototype for pelvic fracture reduction (Bignardi et al., 2018).

Ye et al. (2012) and Wang et al. (Wang et al., 2009; Song et al., 2010) have developed a 6-DOF robot with six independent linear actuators and a stiff end effector supported by two parallel L-shaped tubes, which is expected to provide higher stiffness and positioning accuracy, as shown in Figure 10A. Similarly, inspired by the motorized hexapod-based fracture reduction system proposed by Tang et al. (2012) and Wang et al. (2014) developed a hybrid robot system for reducing long bone fractures, as depicted in Figure 10B. In their subsequent study, Li et al. (2014) developed a robot with a positioning and reduction unit that was used to place fractured legs in the rings, as shown in Figure 10C. Considering the safety factor, Li et al. (2016) proposed a master–slave teleoperation robot suitable for simple fractures, as shown in Figure 10D.

Compared to diaphyseal or long bone fracture reduction, joint fracture and pelvic fracture reduction requires a rather small translational and rotational workspace; however, it requires more accurate repositioning of the fracture fragments. To address these issues, Raabe et al. (2012) used a serial–parallel hybrid robot to reduce intra-articular joint fractures. In their subsequent study, Dagnino et al. (Dagnino et al., 2016b; Dagnino et al., 2016c) developed a hybrid robot using a parallel robot with a UR10 and a position-control method for accurate and repeatable fragment manipulation during minimally invasive joint fracture surgery, as shown in Figure 10E. In 2006, Dagnino et al. (2016a) redesigned the robotic configuration by replacing the UR10 with a carrier platform (CP), as shown in Figure 10F. For a two-fragment joint fracture, Dagnino et al. (Dagnino et al., 2017b; Dagnino et al., 2017c) improved upon their earlier prototype (Dagnino et al., 2016b; Dagnino et al., 2016c) by allowing simultaneous manipulation, as illustrated in Figure 10G. Bignardi et al. (2018) used a hexapod vertically positioned under a surgical table for pelvic fractures, as shown in Figure 10H.

Robots that use hybrid mechanisms combining series and parallel designs can benefit from both types of robots. Consequently, they are becoming a mainstream option and are receiving increasing research attention.

## 5 Discussion

In recent years, RAFR systems have made progress in research on specific fracture types, such as femur, long bone, joint, and pelvic fractures. Possible benefits of the RAFR system include the following.

(1) Improved reduction accuracy, that is, precise alignment of broken fragments and restoration of functionality (Bai et al., 2019; Zhao et al., 2020).
(2) Physical reduction of fractures by accurate and safe robotic assistance while minimizing soft tissue damage, resulting in better clinical outcomes (Zheng et al., 2007; Li et al., 2015a; Zhao et al., 2020).
(3) Reduced physical work by surgeons, such as the large forces required for patient leg traction and maintenance before fixation (Du et al., 2015b; Dagnino et al., 2017b).
(4) Substantial reduction in the cumulative exposure of surgeons to radiation (Westphal et al., 2006; Wang et al., 2014).
(5) Improvement of preoperative planning (Westphal et al., 2009b; Zhao et al., 2022a).
(6) Enhanced intraoperative visualization to understand the 3D fracture configuration better in real-time and reduce the mental burden on surgeons (Westphal et al., 2009a; Dagnino et al., 2016b; Kim and Ko, 2019).
(7) Shortening of the learning curve of skill acquisition (Zhao et al., 2022b; Ge et al., 2022).

### 5.1 Current difficulties in RAFR system research

Most studies on RAFR systems have only reached the mockup experiment or cadaveric experiment stage, and clinical experiments are rare (Dagnino et al., 2017b; Ge et al., 2022). Based on the above review of the main stages and technologies involved in RAFR systems, the major difficulties that may impede further development of RAFR systems for use in clinical applications are as follows.

(1) No universal surgical planning solution exists for achieving high-accuracy virtual reduction for all types of fractures. The accuracy of virtual reduction directly affects the actual fracture reduction accuracy (Cimerman and Kristan, 2007). Surgeons can rely on their personal experiences and use CAD tools to facilitate the process with the help of haptic systems or VR environments. However, this approach can be challenging, time-consuming, and prone to errors. The method of matching the fracture area works only for specific fracture types, such as femur or long-bone fractures. Given the aforementioned situation, some researchers have conducted virtual reduction using a mirror template and achieved satisfactory results. However, this method fails in cases of bilateral fractures.
(2) Realistic surgical workflows and situations are not considered adequately (Dagnino et al., 2017c). Many scholars have followed the clinical workflow of first placing orthopedic pins and then conducting a CT scan and intraoperative registration. However, this procedure cannot satisfy actual clinical requirements as it requires a sterile environment, and typically, no CT or CT-like scanner is available in the operating room. However, real-time tracking of fractured bones assumes a rigid connection between the fractured bone and orthopedic pins, ignoring the deformation of the pins. However, during actual fracture procedures, reduction forces as high as 411 N exist (Gösling et al., 2006), which may cause pin deformation, introduce tracking errors, and result in reduction errors.
(3) Balancing safety and ease of surgery can be challenging. RAFR systems usually employ two types of human–robot interactions to reduce fractures. The first type is an automatic reduction based on a preoperatively planned reduction path, which does not require intervention by the surgeon. This type represents a simple collaborative mode between the surgeon and the robot, reducing the labor intensity of the surgeon. However, this feature is currently limited to simple experimental scenarios in which preoperative planning may not completely align with reality, owing to the human body’s intricate nature and the surgeon’s cognitive abilities. This misalignment can cause collisions and abnormal reduction forces and may even prevent successful reduction. The second type of operation is through master–slave teleoperation, which improves safety but requires a high level of mental labor from the surgeon. The surgeon watches a navigation display during the procedure to control the reduction process.
(4) A risk of secondary injury exists. Surgeons plan reduction paths during preoperative procedures based on their experience and fundamental principles. To ensure a smooth reduction procedure, performing any necessary pre-distraction measures, using the minimum force necessary to move the fragments, and selecting the shortest reduction path to minimize any unnecessary movement is important. However, this process is subjective, and surgeons may increase the distance between bones to avoid collisions, which creates iatrogenic injury and leads to secondary soft tissue injury (Pan et al., 2023). During operative procedures, surgeons using RAFR systems may not be able to feel the forces applied to patients in real time, unlike in other treatments. This lack of tactile feedback can affect their ability to make accurate judgments and may lead to secondary injuries. Although some researchers have installed six-dimensional force sensors at the robotic end effector to monitor the reduction force, determining the threshold limit value remains a challenge (Joung et al., 2010).

### 5.2 Trends and suggestions for future research

After reviewing the technological evolution of RAFR surgical procedures, additional research may be necessary to ensure that the RAFR system is suitable for clinical use. Future research should focus on developing an integrated and practical system that includes automatic preoperative planning and intraoperative safety measures.

#### 5.2.1 Integrated and practical system

Compared with endoscopic surgical robots, which have wide range of applications, RAFR systems are usually designed for particular types of fractures or even specific subclasses. Furthermore, their high cost severely limits their widespread use. In addition, significant gaps remain among mockup experiments, cadaveric experiments, and actual clinical applications. We propose three possible ways to improve the practicality and economy of operation of such systems.

(1) Specialized serial–parallel hybrid robot: A specialized serial–parallel hybrid robot with a high reduction force and torque and a large workspace, without a traction device, may be suitable for many types of fractures and can improve economic considerations.
(2) Auxiliary functions: The reduction procedure is only part of the surgical procedure. The application of RAFR systems can be extended using navigation and robots, such as assisted preoperative orthopedic pins and fixation device implantation.
(3) Fusion-based image registration techniques: The increasing popularity of CBCT in the operating room has enabled the acquisition of intraoperative CBCT data on fractured bones following orthopedic pin implantation in a sterile operating environment, which enables precise and straightforward intraoperative registration with preoperative CT and intraoperative CBCT data during clinical operations, making RAFR systems more advantageous in actual clinical practice.

#### 5.2.2 Automatic preoperative planning

Precise preoperative planning is critical to ensure the effectiveness of reduction operations. Developing computer and imaging technologies has enabled the automation and high accuracy of preoperative planning. We suggest two possible methods to improve the level of automation.

(1) Automatic virtual reduction. SSMs are trained using large amounts of intact skeletal data from humans to perform virtual reduction automatically. A mean shape model is then established for each individual as the template. This method can be used for all types of fractures, facilitates further automation, and reduces time consumption.
(2) Automatic reduction path planning. An accurate and comprehensive biomechanical model of the human body is an effective tool for obtaining muscle stress and strain data. By combining this model with appropriate path-planning algorithms that help avoid collisions and shorten the path, RAFR systems can automatically generate optimal reduction paths without requiring surgical intervention.

#### 5.2.3 Intraoperative safety

Safe surgical procedures are fundamental for the clinical application of RAFR systems and demonstrate their advantages. Biomechanical and sensor technologies, as well as human–robot cooperative modes, can promote safer operations and minimize the possibility of secondary injury. We suggest two main directions for developing safety strategies for RAFR systems.

(1) Real-time monitoring of intraoperative force reduction. Navigation systems can calculate and display real-time reduction forces using biomechanical models of the human body and the real-time positions of the fractured bones or sensors that can be implanted in orthopedic pins. Real-time reduction forces can help alert surgeons to avoid secondary injuries and improve surgical safety.
(2) Human–robot cooperation based on task autonomy. The basic task unit is formed by decomposing the reduction process. By analyzing the strengths and weaknesses of human and robot operations, the dynamic assignment of authority between humans and robots can be realized to control the task unit. This approach not only improves the autonomous operation ability of the robot but also ensures the real-time control ability of the surgeon for operational safety.

## 6 Conclusion

This paper reviews the primary stages of RAFR surgery, including the techniques and methods commonly used over the past decade. Most scholars have developed RAFR systems to treat lower extremity diaphyseal fractures, such as long bone and femoral fractures. They have made considerable progress and achieved high accuracy in fracture reduction, satisfying the clinical requirements. Researchers have recently focused on developing RAFR systems for more complicated fractures, such as joint and pelvic fractures. However, this area is still in the initial stages of development.

The primary stages and key problems remain unchanged regardless of the fracture type. The preoperative planning stage focuses on automatic and accurate planning. A template-based approach has been universally recognized and applied to all types of fractures to calculate the target position of fracture fragments. A 3D model can be used for reduction path planning, and a series of basic principles should be followed, such as maintaining an appropriate distance between fragments, avoiding unnecessary movements, and effectively avoiding collisions and secondary injuries. During the intraoperative stage, the focus is on safely, easily, and accurately performing the surgery. For intraoperative registration, mainstream methods use preoperative CT and intraoperative image data for 2D/3D feature-based, 3D/3D feature-based, or image fusion-based registration. For navigation, a real-time 3D navigation system based on preoperative CT and optical trackers has become an indispensable tool for closed fracture reduction of all types. As a reduction robot, a serial–parallel hybrid robot can provide an excellent balance between workspace and force/torque reduction, making it a better choice. In addition, the significance of human–robot cooperation for surgical safety lies in fully utilizing both humans and robots.

Currently, RAFR systems are primarily in the laboratory stage and far from commercialization owing to unresolved difficulties. In the future, RAFR systems will evolve into integrated and practical systems with automatic preoperative planning and high intraoperative safety, further promoting their development.
